# Gradient V-Shaped and N-Shaped Seismic Metamaterials

**DOI:** 10.3390/ma16083074

**Published:** 2023-04-13

**Authors:** Yu-Chi Su, Sheng-Shiang Wang

**Affiliations:** Department of Civil Engineering, National Cheng Kung University, Tainan 70101, Taiwan

**Keywords:** seismic metamaterial, surface wave, bandgap, wave attenuation, seismic wave

## Abstract

Seismic metamaterials provide an innovative alternative in earthquake engineering by reducing the hazards from seismic waves without modifying the existing structures. Although many seismic metamaterials have been proposed, a design for a broad bandgap at low frequencies is still in demand. In this study, two novel seismic metamaterials, V- and N-shaped designs, are proposed. We found that by adding a line to the letter V, turning the V-shaped design into an N-shaped design, the bandgap can be broadened. Both the V- and N-shaped designs are arranged in a gradient pattern to combine the bandgaps from metamaterials with different heights. Using only concrete as the base material for the design makes the proposed seismic metamaterial cost effective. Finite element transient analysis and band structures are in good agreement, validating the accuracy of the numerical simulations. Surface waves are effectively attenuated over a broad range of low frequencies using the gradient V- and N-shaped seismic metamaterials.

## 1. Introduction

Traditional seismic-resistant technology, such as structural reinforcements and energy dissipation systems, is embedded in structures [1]. Seismic metamaterials provide a novel approach to mitigate seismic waves without retrofitting existing structures. Several types of seismic metamaterials, such as structured soil, buried mass resonators, above surface resonators, and auxetic material are proposed in the literature [2,3]. The pioneer work of seismic metamaterials begins with the category of structured soil. Richart et al. [4] proposed the idea of shielding from seismic waves using cylindrical holes. Meseguer et al. [5] experimented with the efficiency of surface wave attenuation from cylindrical holes in honeycomb and triangular lattices. Kattis et al. [6] examined the screening performance of concrete pile rows using an effective trench model. Brûlé et al. [7] performed a large-scale experiment on vertical empty inclusions bored into the soil. Achaoui et al. [8] proposed the design of periodic columns clamped to underlying bedrock to create a zero-frequency bandgap. Du et al. [9] compared the concrete-filled pile with hollow cylinder, rectangular, and square shaped piles and found that the concrete-filled pile gives the most desirable performance. Li et al. [10] embedded radially periodic steel rings into soil to attenuate seismic waves. Miniaci et al. [11] proposed a cross-like cavity model and compared it with hollow and coated cylinders. Zhang et al. [12] proposed four seismic metamaterial models for Lamb waves using cross-like and square steel sections. The square shape enhances the performance of the seismic metamaterial compared to the cross-like steel section. Varma et al. [13] studied the inclusion of steel shapes configured as square, circular, and notch-shaped square. They found that the square steel configuration is superior to the other two under the same filling ratio. The clamped boundary can also significantly shift the bandgap to lower frequencies.

For the category of buried mass resonators, Finocchio et al. [14] proposed a model composed using a sphere rolling over a cycloidal trajectory to attenuate S waves. Krödel et al. [15] conducted an experiment on soil-embedded cylindrical tubes containing a resonator. Palermo et al. [16] developed a model that consists of a cylindrical mass suspended using elastomeric springs within a concrete case. They also utilized effective medium theory to study the mode conversion mechanism. Achaoui et al. [17] numerically investigated a model of iron spheres with attached ligaments embedded into concrete. Palermo et al. [18] applied multi-mass resonators in a seismic metamaterial to mitigate surface waves. Xu et al. [19] proposed a design using H-shaped steel columns wrapped with rubber to generate a wide bandgap. Wang et al. [20] coated a concrete square pipe with rubber and embedded it into soil as a two-dimensional seismic metamaterial with an ultralow frequency bandgap. Seismic metamaterials composed of the same base material such as steel and aluminum are prevalent in the category of above-surface resonators. Colquitt et al. [21] investigated the cylindrical seismic metamaterials on a thin elastic plate and an elastic half space. Zeng et al. [22] designed an I-shaped pillar as a seismic metamaterial and compared it with cylindrical and rectangular pillars. Muhammad et al. [23] applied built-up steel sections to create seismic metamaterials for surface wave mitigation. Xu et al. [24] proposed a cylinder design with a cross-shaped steel running through its length. Zeng et al. [25] proposed an inverted T-shaped seismic metamaterial. Ji and Yu [26] evaluated the performance of the I-shaped, T-shaped, inverted T-shaped, and simple rod seismic metamaterials and concluded that the T-shaped design results in the broadest bandwidth. The use of metals gives a broad bandwidth, but at the same time, increases the cost. Instead of metal, Su and Wu [27] designed the snowman-like seismic metamaterial only using concrete. Using more than one material, Chen et al. [28] proposed a ternary seismic metamaterial composed of rubber, steel, and soil. Zeng et al. [29] designed the above-surface resonator comprising a steel block wrapped with foam, making it easy to manufacture. Miniaci et al. [30] used self-similar cross-like cavities to demonstrate how a hierarchical design improves the capability of seismic metamaterials. Forest seismic metamaterials are also classified as one type of above-surface resonators [31,32,33]. Although there have been several auxetic metamaterials proposed in the literature [34], applying them in earthquake protection is a novel development. Ungureanu et al. [35] proposed the idea that the auxetic seismic metamaterial could be used in earthquake engineering as an effective alternative to merely increasing the strength of the structural elements. Huang et al. [36] used auxetic foam for the stiffness of resonators to block Lamb waves.

Although a variety of seismic metamaterials have been proposed, most of the designs only generate a narrow bandgap. The need remains for a design with low-frequency bandgap and broad bandwidth. Much effort has been put into achieving this. Colombi et al. [37] combined graded and resonant structures to develop a metawedge for trapping or converting surface waves. Du et al. [38] designed H-fractal seismic metamaterials with different levels to widen the bandwidth. Zeng et al. [39] proposed a design based on a Matryoshka-like structure. They found that a wider bandgap can be achieved by adding layers of the Matryoshka-like design. Liu et al. [40] proposed partially embedded gradient rods. The bandwidth is broadened by combining the bandgaps from rods embedded at different lengths. Wu et al. [41] combined above-ground pillars and embedded core-shell units to form a seismic metamaterial design to widen the bandgap. Zeng et al. [42] numerically and experimentally demonstrated that inertial amplification can be used in seismic metamaterial design for isolating surface waves. It is noted that there are other potential applications for metamaterials [43,44].

In this study, V- and N-shaped novel seismic metamaterials are proposed. While the V-shaped design adequately blocks seismic waves in a certain frequency range, adding a line to the letter V, converting it to an N-shaped seismic metamaterial, improves the bandgap. Arranging both designs in a gradient pattern further broadens the respective bandwidth of each. Adding lines to V geometry can be applied to create most English alphabet shaped designs, and the gradient configuration can effectively broaden their bandgaps. Results from transient wave analysis match the band structure, verifying the accuracy of numerical simulations. This study proposes effective ways for seismic metamaterials to broaden low-frequency bandgaps at low costs.

## 2. V-Shaped and N-Shaped Models

**Figure 1:**
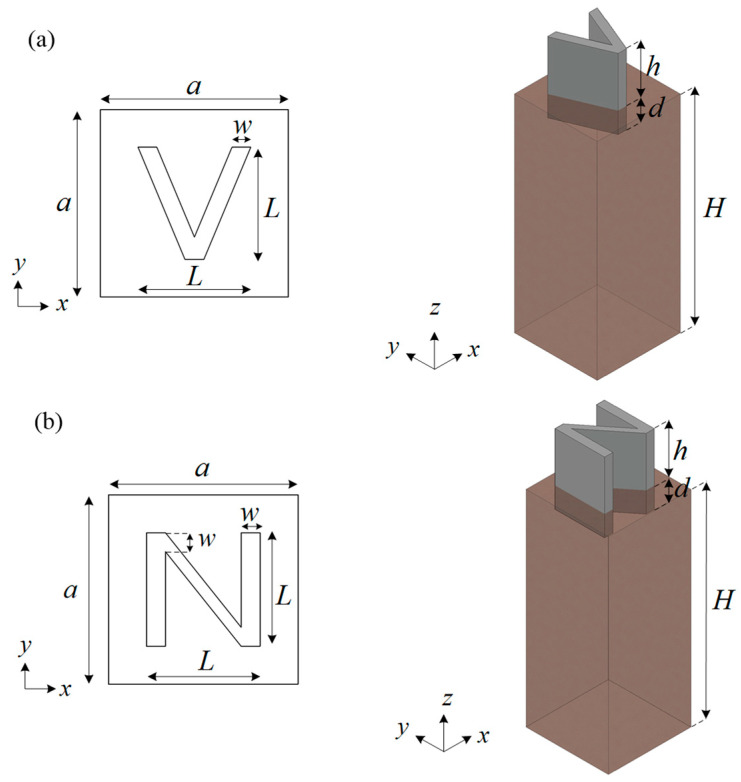
(**a**) V-shaped seismic metamaterial; (**b**) N-shaped seismic metamaterial.

Figure 1 and Table 1 show the V-shaped and N-shaped seismic metamaterials proposed in this study. In Figure 1, the gray and brown colors represent concrete and soil, respectively. Considering surface waves, each design is arranged in a square lattice on a half space with lattice constant a=2 m. As a gradient pattern, the height of the seismic metamaterials varies from h=1.2 m to h=2.4 m, and each metamaterial is embedded into soil with d=0.5 m to increase stability. The depth of soil is set to H=100 m to simulate a half space. To demonstrate the band structures and wave attenuation of the V-shaped and N-shaped seismic metamaterials, the finite element software COMSOL Multiphysics is used.

## 3. Methods of Simulations

### 3.1. Band Structures

**Figure 2:**
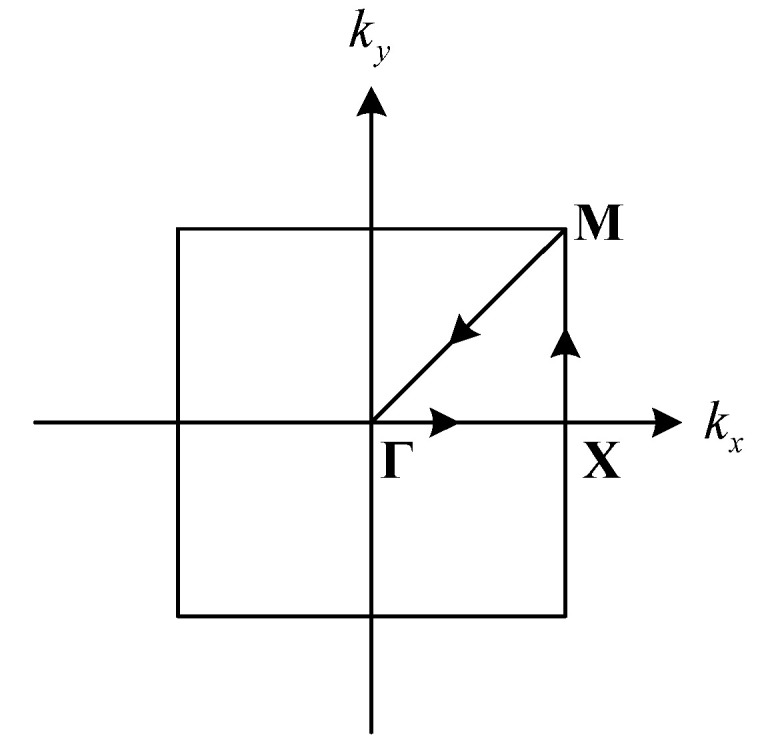
First irreducible Brillion zone for a square lattice: Γ=(0,0), X=(π/a,0), M=(π/a,π/a).

Based on the Bloch theorem [45], the band structure of an infinite periodic structure can be reduced to a unit cell with periodic boundary condition:

(1) $$u(r+a,t)=u(r,t)e^{ik\cdot a},$$

where u is the displacement of the periodic structure, a is the lattice vector, and k is the wave vector in the first Brillion zone [46]. Therefore, for calculating the band structures of V- and N-shaped seismic metamaterials, the periodic boundary conditions are applied on the lateral sides of the unit cells in Figure 1. Traction free and fixed boundary conditions are used at the top and bottom of the unit cell, respectively. Eigenfrequency study in the solid mechanics module is used in the finite element software COMSOL Multiphysics. Sweeping the wave vector along the first irreducible Brillion zone, Γ→X→M→Γ, as shown in Figure 2, the band structure of the model can be obtained.

### 3.2. Surface Wave Attenuation

**Figure 3:**
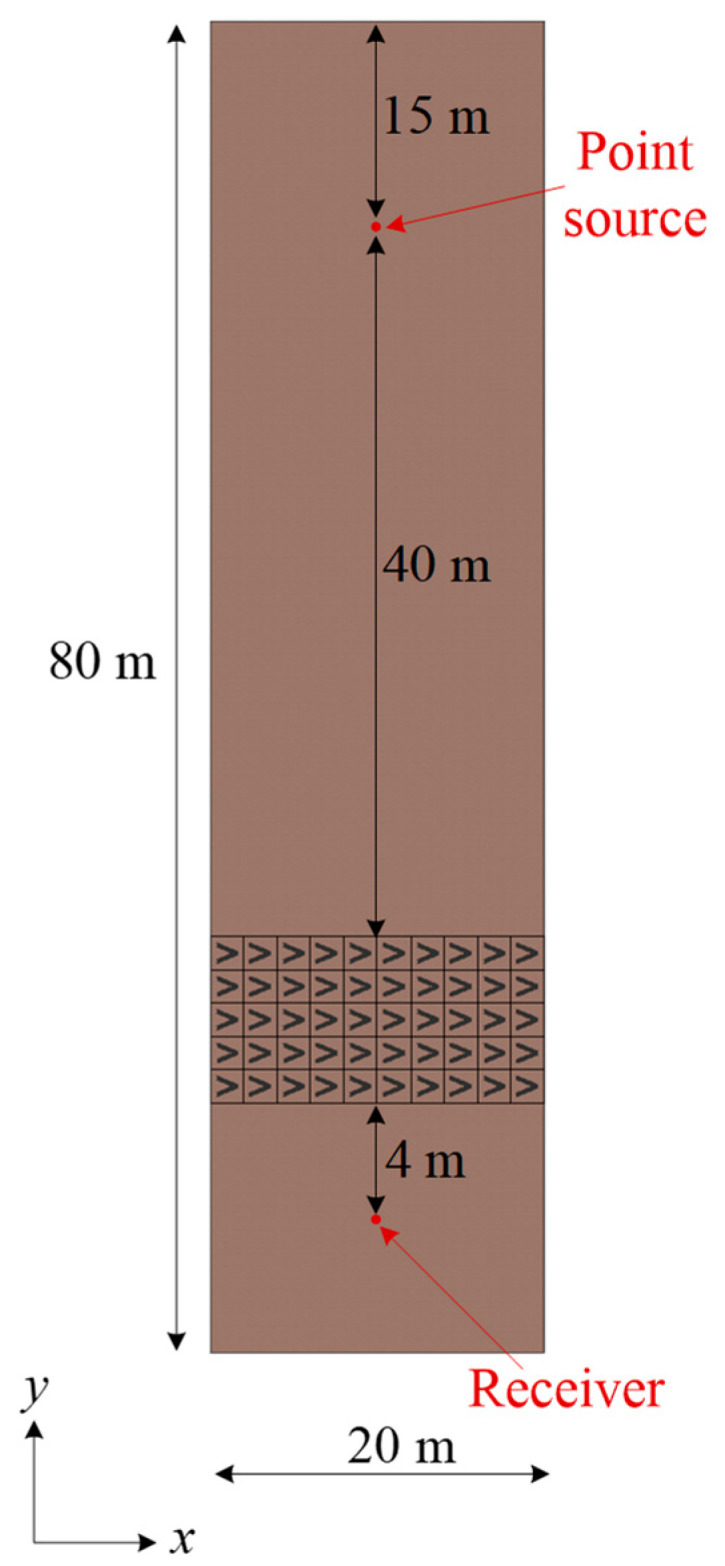
Configuration of the transient simulation.

To validate the performance of V and N shaped seismic metamaterials, transient analysis is performed. Time-dependent study in the solid mechanics module is used in the finite element software COMSOL Multiphysics. Taking the V-shaped model, for example, a 20 m×80 m×100 m soil slab is built, as shown in Figure 3. Low reflecting boundaries are applied on the lateral sides and the bottom of the slab to simulate a half space. The free tetrahedral elements with quadratic serendipity shape functions are used for transient simulations. A Gaussian wave packet is utilized as an out-of-plane displacement point source covering the frequency region of a bandgap as follows:

(2) $$u_{z}=u_{0}e^{-\frac{{\left(t-t_{0}\right)}^{2}}{T_{0}^{2}}}\sin(2\pi f_{0}(t-t_{0})),$$

where $u_{0}$ is the displacement amplitude, $f_{0}$ is chosen as the averaged frequency of the bandgap region, and $T_{0}$ is the parameter controlling the bandwidth of the Gaussian wave packet. The time shift for the Gaussian wave packet is determined by parameter $t_{0}$.

## 4. Results and Discussion

### 4.1. Band Structures

**Figure 4:**
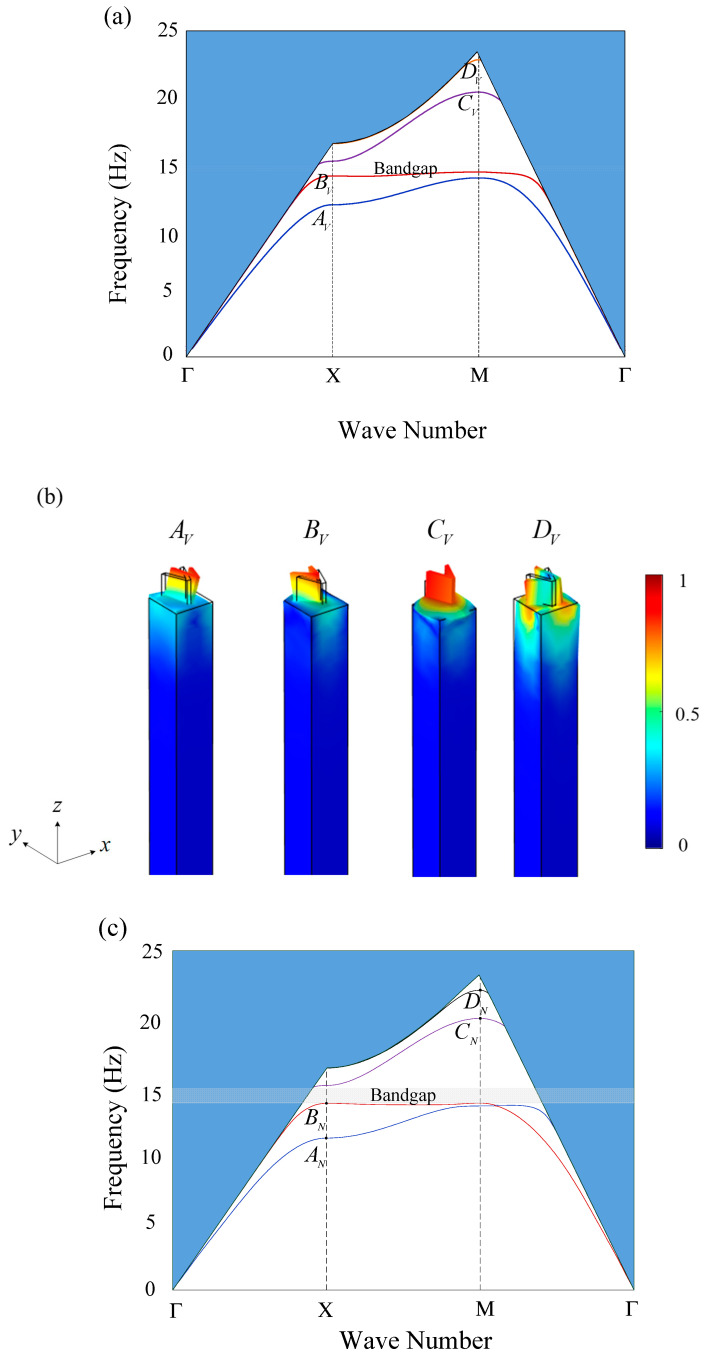

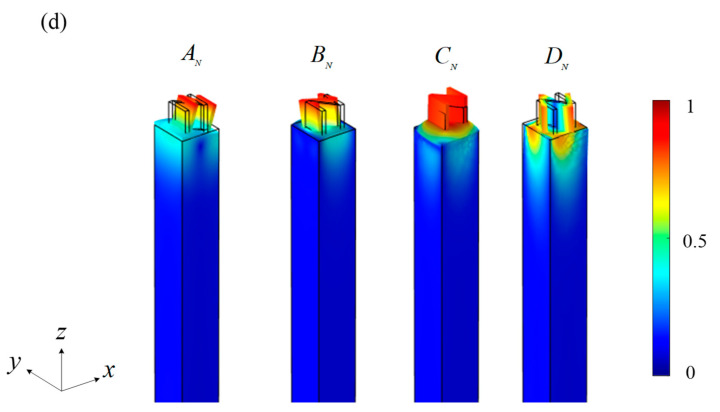
(**a**) Band structure of the V-shaped model. The first bandgap is 14.18–14.72 Hz; (**b**) Vibration modes for the V-shaped model corresponding to the band structure; (**c**) Band structure of the N-shaped model. The first bandgap is 13.77–14.80 Hz; (**d**) Vibration modes for the N-shaped model corresponding to the band structure.

For comparison, h=1.2 m is chosen to demonstrate band structures. The other geometric and material parameters are described in Section 2. The band structures and the corresponding vibration modes for the V and N shaped seismic metamaterials are shown in Figure 4a–d. In Figure 4a,c, the blue regions indicate the sound cone [47] which differentiate surface waves from the bulk waves. The first and second passing bands are the tilting modes, as shown in Figure 4b,d. Since the moment of inertia in the x and y directions are not equal, these two passing bands do not overlap. The third passing band is the elongational mode, and finally, the fourth passing band is the rotational mode. Both first bandgaps, as shown in Figure 4a,c, start from the tilting mode and end at the elongational mode. The bandgap region for the V-shaped model is 14.18–14.72 Hz, while the N-shaped design is 13.77–14.80 Hz. The N-shaped seismic metamaterial improves the first bandgap of the V-shaped design. The reason for this phenomenon can be observed in the lower and upper bounds of the bandgap. For the lower bound, since the volume of the N-shaped model is larger than the V-shaped model, the N-shaped model has a larger moment of inertia. A larger moment of inertia lowers the natural frequencies of the tilting modes. For the upper bound of the bandgap, the elongational stiffness EA/L is higher if the model has a larger cross-sectional area A. Since the N-shaped model is made by adding a line to the V-shaped model, its cross-sectional area is larger. As a result, a higher natural frequency of the elongational mode is achieved. Thereby, the N-shaped seismic metamaterial has a broader bandgap than the V-shaped model by increasing the moment of inertia and the elongational stiffness.

### 4.2. Parametric Studies

**Figure 5:**
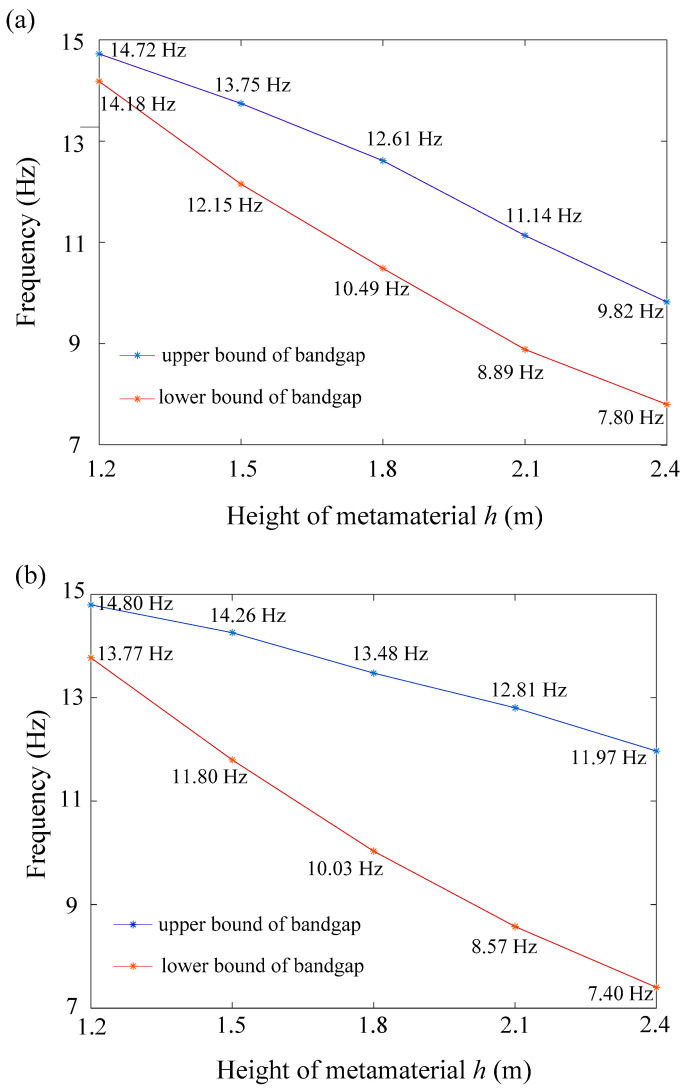
(**a**) Effect of height on the first bandgap of the V-shaped model; (**b**) Effect of height on the first bandgap of the N-shaped model.

To investigate the effects of metamaterial height, parametric studies are performed. As shown in Figure 5a, the above surface heights of the V-shaped seismic metamaterial are set to be 1.2 m, 1.5 m, 1.8 m, 2.1 m, and 2.4 m, respectively. Band structure simulations corresponding to these five different heights are performed. The results show that the lower bound of the bandgap significantly decreases as the height h increases. This is due to taller metamaterials having a higher center of mass, which in turn shifts their tilting modes to lower frequencies. The upper bound of the bandgap also decreases as the height increases because the increment of height decreases the elongational stiffness, resulting in a lower elongational resonance. The bandwidth is broadened when the metamaterial is taller. Figure 5b shows the parametric study result for the N-shaped model. The value of height h is set to be 1.2 m, 1.5 m, 1.8 m, 2.1 m, and 2.4 m. Similar to the V-shaped model, both the lower and upper bounds shift to lower frequencies as the height increases because of a higher center of mass and lower elongational stiffness. The bandwidth is remarkably broadened with each height increment. In the design of gradient V and N shaped seismic metamaterials, we combine the resulting bandgap of each model’s diverse height to generate a broad bandgap for seismic wave mitigation.

### 4.3. Surface Wave Attenuation

**Figure 6:**
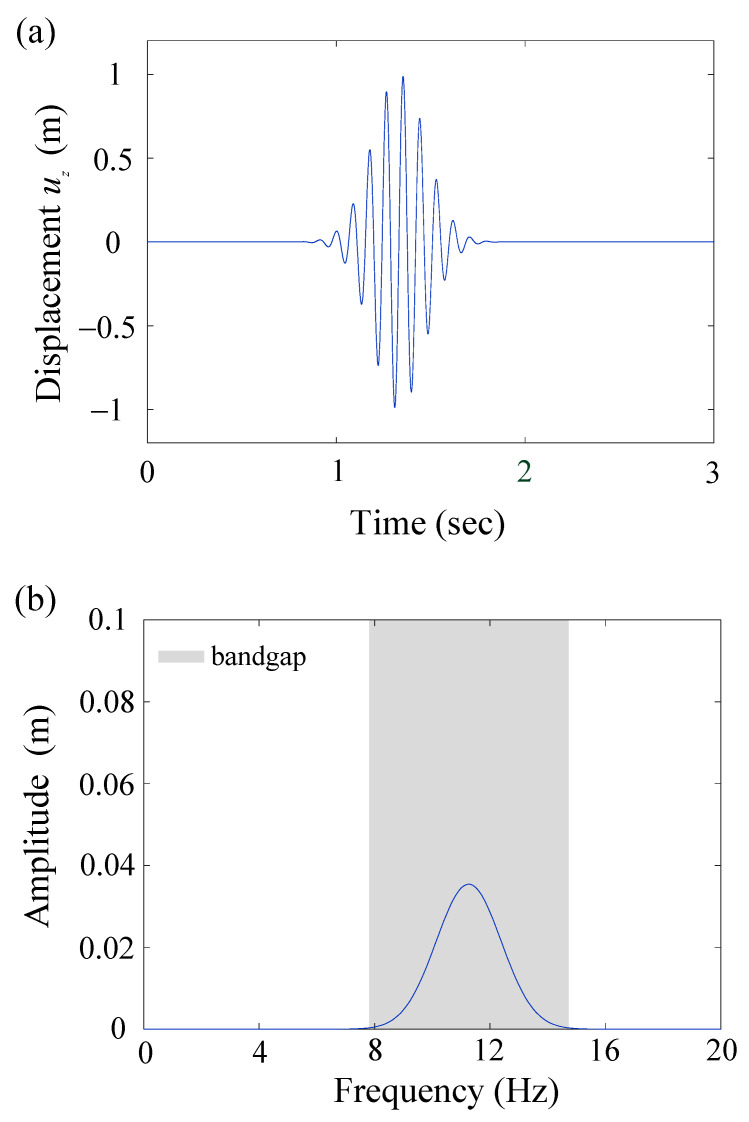

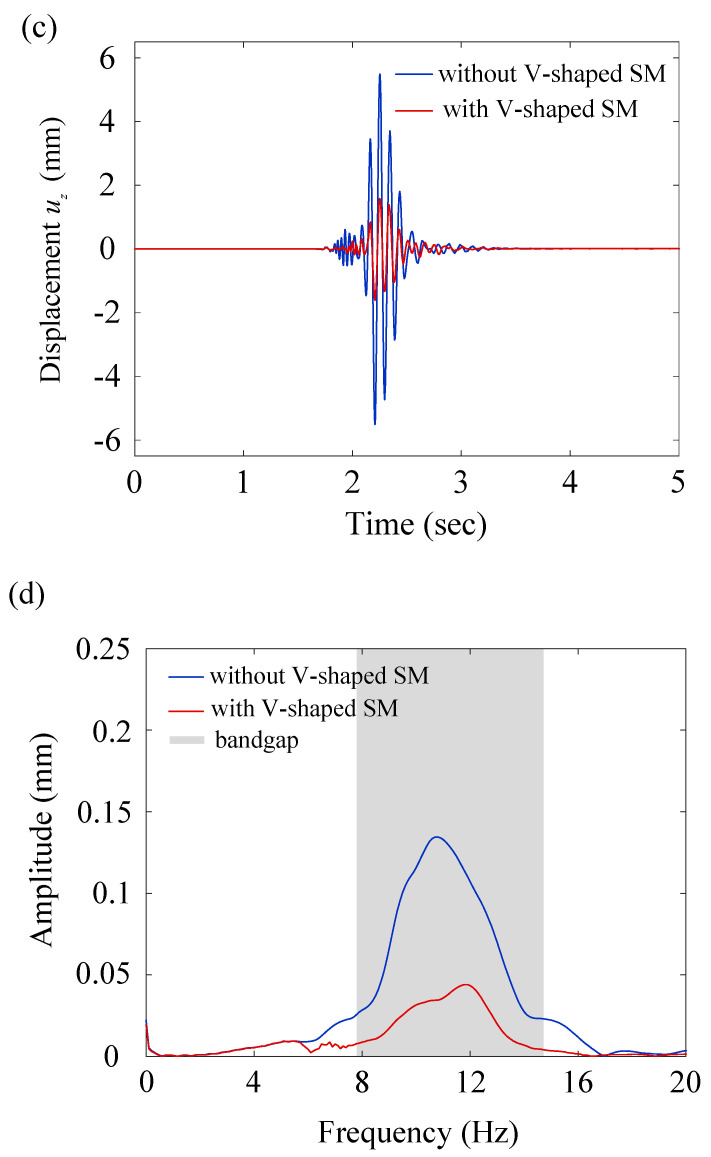
(**a**) Time history of the Gaussian wave packet; (**b**) Frequency contents of the Gaussian wave packet; (**c**) Transient simulation results. Red line indicates the time domain result with the V-shaped seismic metamaterials, while the blue line indicates the result without the V-shaped seismic metamaterials; (**d**) Results in the frequency domain obtained from the Fast Fourier Transform. The gray area indicates the bandgap region from the band structure. All figures are for the gradient V-shaped model.

As shown in Figure 3, there is a square lattice array composed of five rows. Each row contains ten-unit cells of equal height V-shaped seismic metamaterials. These seismic metamaterials are placed at a distance of approximately two to three wavelengths from the point source. The receiver point is set to double the distance of the lattice constant from the seismic metamaterials. A gradient height of the V-shaped model is used for these five rows as 1.2, 1.5, 1.8, 2.1, and 2.4 m. We set higher V-shaped models to be closer to the receiver point. The inverse height variation gives similar results (see Supplementary Material S2). In this case, $u_{0}=1\;m$, $t_{0}=1.33\;s$, $T_{0}=0.20\;s$, and $f_{0}=11.26\;\mathrm{Hz}$ are chosen for Equation (2). The time history and frequency contents of this Gaussian wave packet are shown in Figure 6a,b, respectively. The frequency contents fit the first bandgap region of the gradient V-shaped seismic metamaterial (7.80–14.72 Hz). Figure 6c,d show the results of transient analysis. Red and blue lines indicate the responses with and without V-shaped seismic metamaterials. From Figure 6c, the maximum amplitude 5.5 mm decreases to 1.6 mm when using the gradient V-shaped seismic metamaterial. The amplitude reduction rate is 70.77%. The frequency response obtained from applying Fast Fourier Transform on the time response is shown in Figure 6d. The gray area indicates the bandgap region calculated from the band structure. Comparing the cases with and without seismic metamaterials, the magnitudes are effectively attenuated in the bandgap region. Because the point source excites different waves, and surface waves only receive part of the energy, the magnitudes in Figure 6c,d are smaller than Figure 6a,b, respectively.

**Figure 7:**
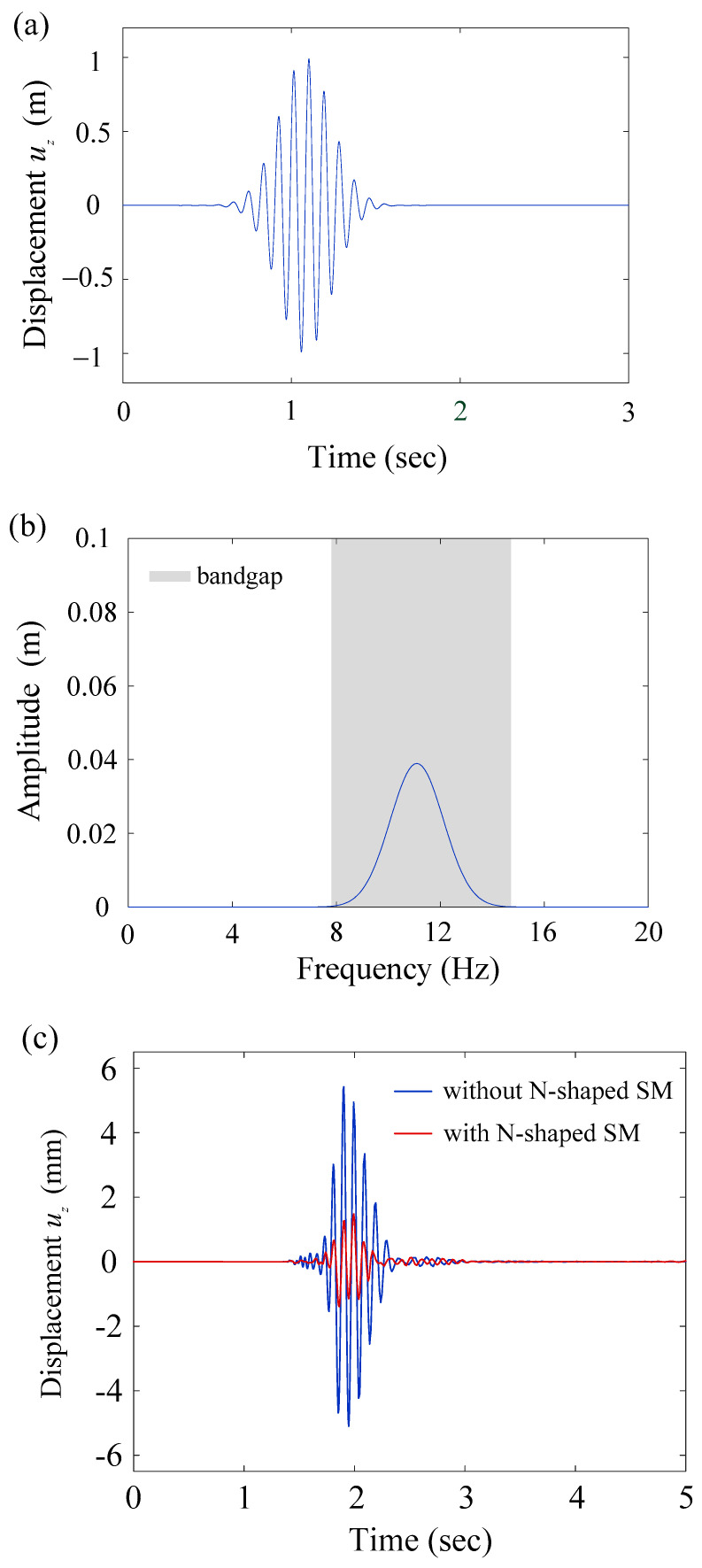

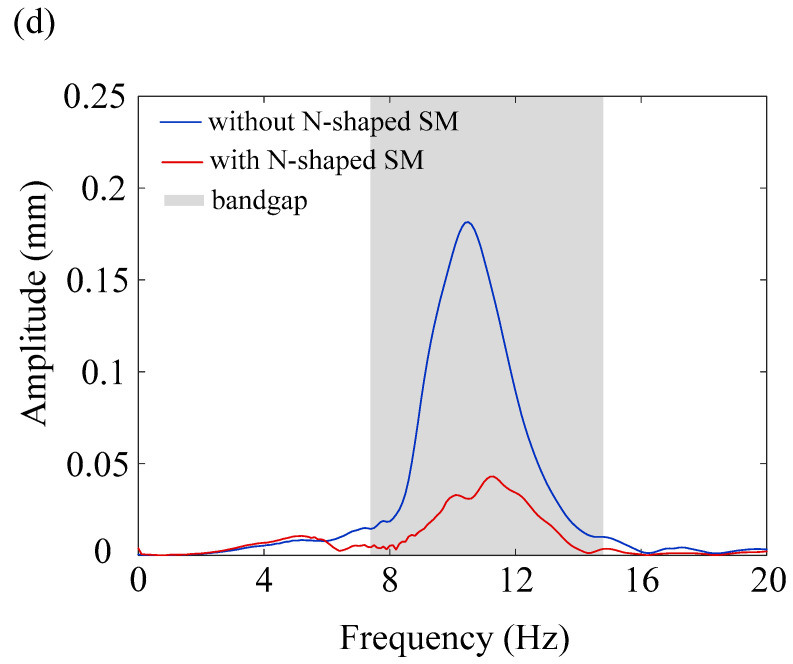
(**a**) Time history of the Gaussian wave packet; (**b**) Frequency contents of the Gaussian wave packet; (**c**) Transient simulation results. Red line indicates the time domain result with the N-shaped seismic metamaterials, while the blue line indicates the result without the N-shaped seismic metamaterials; (**d**) Results in the frequency domain obtained from the Fast Fourier Transform. The gray area indicates the bandgap region from the band structure. All figures are for the gradient N-shaped model.

The geometric configuration of the transient simulation for the gradient N-shaped model is identical to Figure 3, except replacing the V-shaped models with the N-shaped models. The height variation for the gradient N-shaped model remains 1.2, 1.5, 1.8, 2.1, and 2.4 m. The parameters for the Gaussian wave packet are $u_{0}=1$ m, $t_{0}=1.08$ s, $T_{0}=0.22$ s, and $f_{0}=11.10$ Hz. Its time history and frequency contents are shown in Figure 7a,b, respectively. To evaluate the wave attenuation, the frequency contents of the Gaussian wave packet fit the first bandgap region from the band structure. The results are shown in Figure 7c,d. The maximum amplitude 5.4 mm is reduced to 1.5 mm by placing gradient N-shaped seismic metamaterials, as shown in Figure 7c. The amplitude reduction rate is 72.22%. For the frequency response, the magnitudes are all decreased in the bandgap region (gray area), validating the correctness of the numerical simulations. 

## 5. Conclusions

This study proposes V-shaped and N-shaped seismic metamaterials. The N-shaped design has a lower and broader bandgap than the V-shaped design due to a larger moment of inertia and elongational stiffness. By arranging both models in a gradient pattern, the bandgap is considerably broadened. From transient simulations, surface waves are significantly attenuated in the bandgap regions, validating the performance of the V and N shaped seismic metamaterials. The use of concrete as the sole construction material makes the proposed seismic metamaterials more cost-effective. This study provides effective ways for designing low-cost seismic metamaterials with broad and low-frequency bandgaps.

## Figures and Tables

**Table 1:** Geometric parameters and material properties of the V- and N-shaped models.

Geometric Parameters	Concrete [16]	Soil [12]
a=2 m w=0.2 m L=1.2 m	E=30 GPa $\rho =2500\;{\mathrm{kg}/m}^{3}$ υ=0.25	E=20 MPa $\rho =1800\;{\mathrm{kg}/m}^{3}$ υ=0.3

## Data Availability

The data presented in this study are available on request from the corresponding author.

## Supplementary Materials

The following supporting information can be downloaded at: https://www.mdpi.com/article/10.3390/ma16083074/s1, Figure S1. (a) Effect of width on the bandgaps of the V-shaped model; (b) Effect of length on the bandgaps of the V-shaped model; Figure S2. (a) Effect of width on the bandgaps of the N-shaped model; (b) Effect of length on the bandgaps of the N-shaped model; Figure S3. (a) Transient response of the constant height V-shaped model; (b) Frequency spectrum of the constant height V-shaped model; (c) Transient response of the gradient V-shaped model; (d) Frequency spectrum of the gradient V-shaped model; Figure S4. (a) Transient response of the increasing and decreasing height arrangements of the V-shaped model; (b) Frequency spectrum of the increasing and decreasing height arrangements of the V-shaped model.
