# Supplementary material for: Gradient V-Shaped and N-Shaped Seismic Metamaterials
Source: Materials (Basel). 2023 Apr 13;16(8):3074. doi: 10.3390/ma16083074 (PMC10145495; doi:10.3390/ma16083074)
Supplement: Supplementary file 1 [file materials-16-03074-s001.zip › materials-2212745-supplementary.pdf]

Supplementary Material I: Parametric studies on width and length of the V and N shaped models.

Figure S1(a) shows the parametric study on the width of the V-shaped seismic metamaterial. Increasing the width results in a shift of the bandgaps towards lower frequencies. Specifically, when the width reaches 1.3m, the second bandgap appears below 18 Hz. Figure S1(b) shows the parametric study on the length of the V-shaped model. As the length increases, the bandgap shifts to higher frequencies.

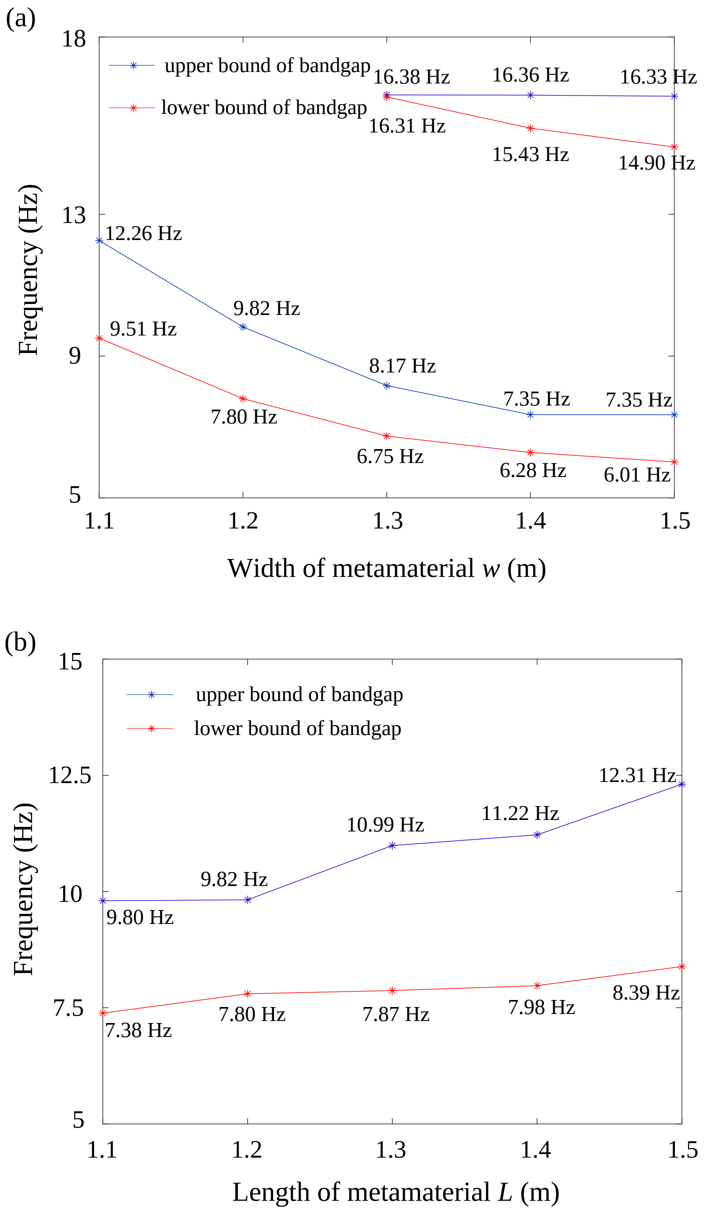

Figure S1 (a) Effect of width on the bandgaps of the V-shaped model; (b) Effect of length on the bandgaps of the V-shaped model.

Similar trends can be found for the N-shaped model, as shown in Figures S2(a) and S2(b).

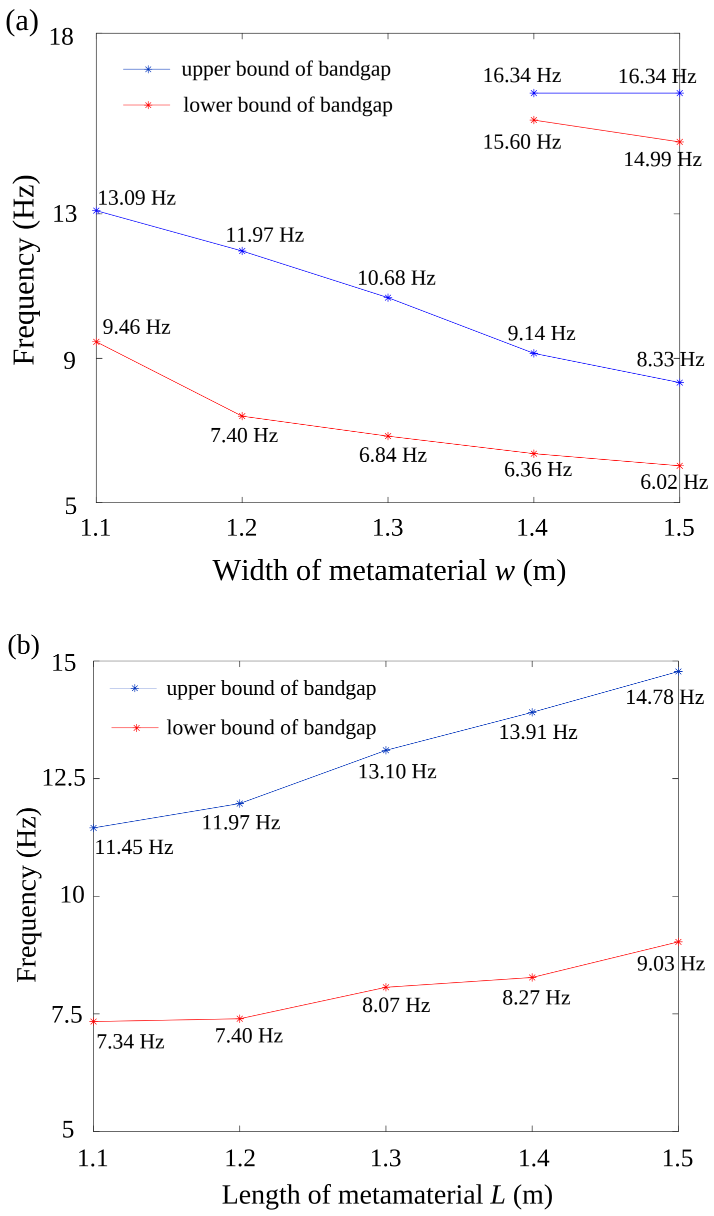

Figure S2 (a) Effect of width on the bandgaps of the N-shaped model; (b) Effect of length on the bandgaps of the N-shaped model.

Supplementary Material II: Studies on height distribution in transient simulations

The transient response and its corresponding frequency spectrum for the constant height V-shaped model are shown in Figures S3(a) and S3(b), respectively. For the constant height V-shaped model, we used $h = 1.2\ m$, and the other simulation settings are the same as Figure 3. The simulation results for the gradient V-shaped model, Figures 6(c) and 6(d), are replotted here as Figures S3(c) and 3(d). Compared Figures 3(a) and 3(c), we found that the gradient model shows better performance in wave attenuation. Observing from frequency domain, as shown in Figures S3(b) and S3(d), it is evident that the gradient model can attenuate waves in a broader frequency region (7.80-14.72 Hz). Therefore, the gradient V-shaped model outperforms the constant height V-shaped model.

(a)

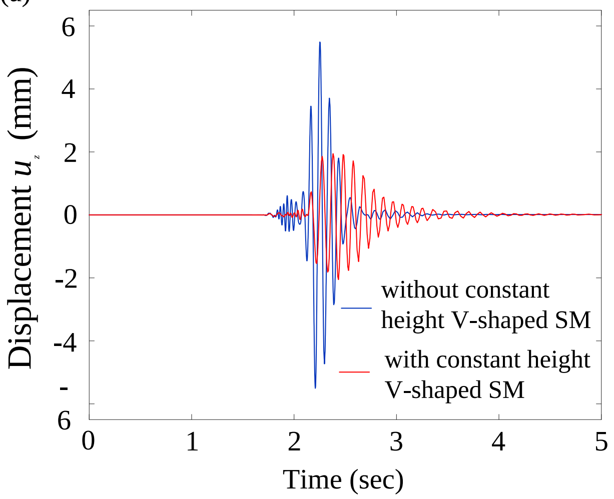

(b)

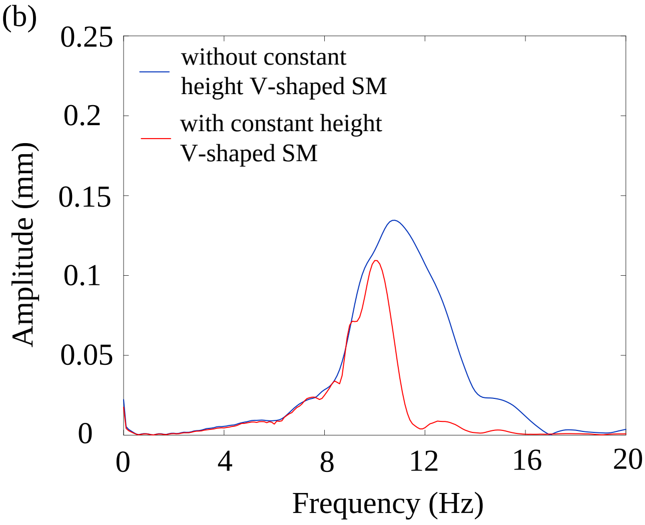

(c)

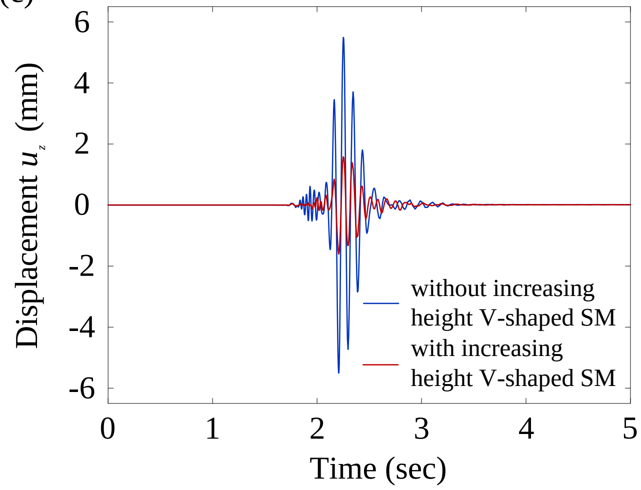

(d)

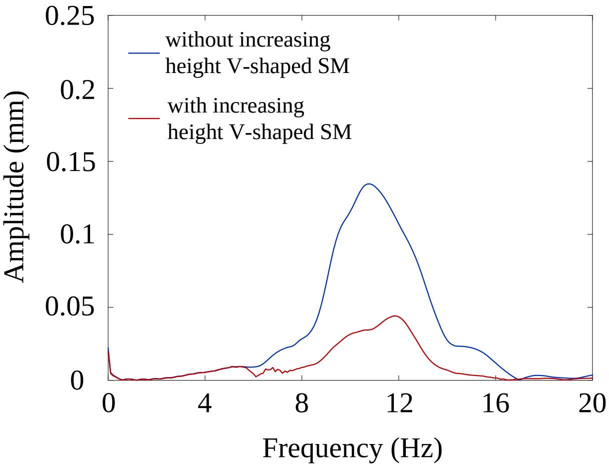

Figure S3 (a) Transient response of the constant height V-shaped model; **(b)** Frequency spectrum of the constant height V-shaped model; **(c)** Transient response of the gradient V-shaped model; **(d)** Frequency spectrum of the gradient V-shaped model.

Figure S4 shows the simulation results for the increasing and decreasing height arrangements of the V-shaped model. The arrangement we selected for the main text is the increasing height V-shaped model. Observing Figure S4, we noted minor differences in time and frequency domains between the two arrangements. Additionally, these two arrangements can attenuate surface waves in the bandgap region.

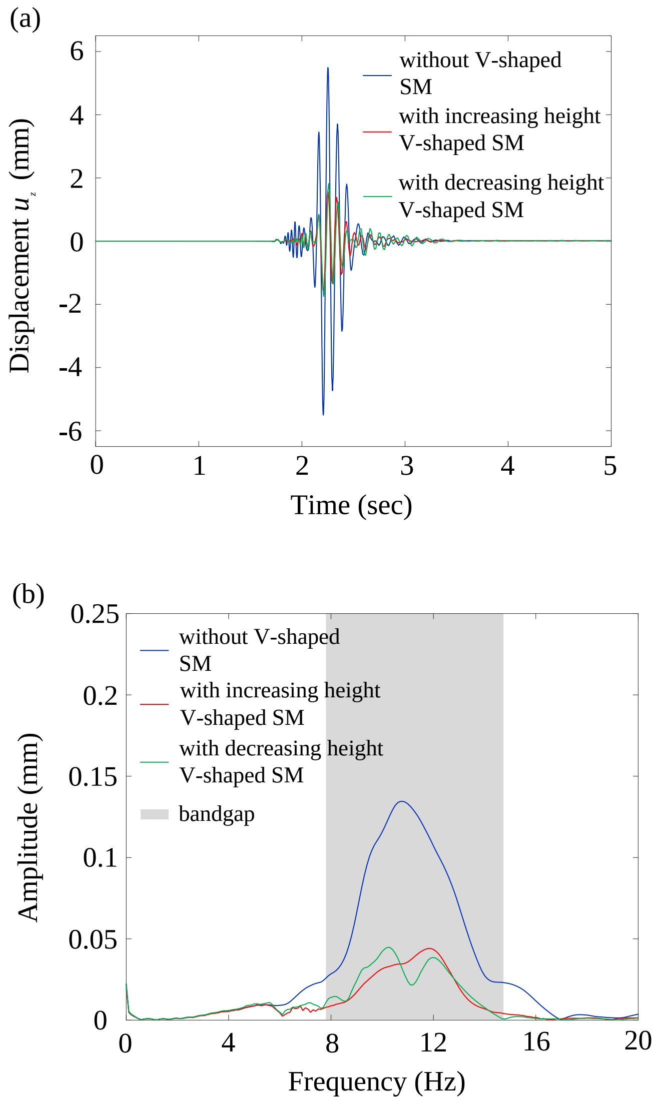

Figure S4 (a) Transient response of the increasing and decreasing height arrangements of the V-shaped model; **(b)** Frequency spectrum of the increasing and decreasing height arrangements of the V-shaped model.
